# Association of *Fasciola hepatica* Infection with Liver Fibrosis, Cirrhosis, and Cancer: A Systematic Review

**DOI:** 10.1371/journal.pntd.0004962

**Published:** 2016-09-28

**Authors:** Claudia Machicado, Jorge D. Machicado, Vicente Maco, Angelica Terashima, Luis A. Marcos

**Affiliations:** 1 Cancer Genomics and Epigenomics Laboratory, Department of Cellular and Molecular Sciences, School of Sciences and Philosophy, Universidad Peruana Cayetano Heredia, Lima, Peru; 2 Institute for Biocomputation and Physics of Complex Systems, University of Zaragoza, Spain; 3 Division of Gastroenterology, Hepatology and Nutrition, University of Pittsburgh Medical Center, Pittsburgh, Pennsylvania, United States of America; 4 Laboratorio de Parasitologia, Instituto de Medicina Tropical Alexander von Humboldt, Universidad Peruana Cayetano Heredia, Lima, Peru; 5 Division of Infectious Diseases, Department of Medicine, Stony Brook University, Stony Brook, New York, United States of America; Department of Molecular Genetics and Microbiology, Stony Brook University, Stony Brook, New York, United States of America; Universidad Peruana Cayetano Heredia, PERU

## Abstract

**Background:**

Fascioliasis has been sporadically associated with chronic liver disease on previous studies. In order to describe the current evidence, we carried out a systematic review to assess the association between fascioliasis with liver fibrosis, cirrhosis and cancer.

**Methodology and Principal Findings:**

A systematic search of electronic databases (PubMed, LILACS, Scopus, Embase, Cochrane, and Scielo) was conducted from June to July 2015 and yielded 1,557 published studies. Among 21 studies that met inclusion and exclusion criteria, 12 studies explored the association of *F*. *hepatica* with liver fibrosis, 4 with liver cirrhosis, and 5 with cancer. Globally these studies suggested the ability of *F*. *hepatica* to promote liver fibrosis and cirrhosis. The role of *F*. *hepatica* in cancer is unknown. Given the heterogeneity of the studies, a meta-analysis could not be performed.

**Conclusions:**

Future high-quality studies are needed to determine the role of *F*. *hepatica* on the development of liver fibrosis, liver cirrhosis, and cancer in humans.

## Author Summary

Fascioliasis is a neglected infectious disease caused by the trematode *Fasciola*. This parasite (liver fluke) is endemic in many parts of the world including countries from Asia, Africa, Europe and the Americas. High prevalence rates of fascioliasis has been repeatedly reported in the Andean region of South America. Liver complications in infected humans by this fluke have been sporadically reported in the literature. For instance, the relationship between *F*. *hepatica* infection and liver fibrosis has been suggested but its association with cancer is unclear. In this study, we found some evidence of an association between *Fasciola* infection with liver fibrosis and cirrhosis, but little between *Fasciola* and cancer. As *Fasciola* is highly endemic in some regions of the developing world, our study shed light on the complications of this parasitic infection which are not that different from flukes such as *Schistosoma* or *Opisthorchis*. We believe that further investigations are needed in order to elucidate the pathways in how *F*. *hepatica* infection causes liver damage.

## Introduction

Food-borne trematodiases are an emerging public health problem in Southeast Asia and Latin America, and are caused by the following flukes: *Clonorchis sinensis*, *Fasciola gigantica*, *Fasciola hepatica*, *Opisthorchis felineus*, *Opisthorchis viverrini*, and *Paragonimus spp* [1]. Globally, it has been estimated that approximately 56 million people are infected by these parasites [2]. According to the International Agency for Research on Cancer, two of these parasites (*O*. *viverrini* and *C*. *sinensis*) have been recognized as definitive causes of cancer [3]. However, fascioliasis caused by *F*. *hepatica* or *F*. *gigantica* has not been clearly associated with cancer to date.

Fascioliasis, as a neglected tropical disease, commonly affects poor people from developing countries [4]. It has been estimated that at least 2.6 million people are infected with fascioliasis worldwide [2]. When a combination of serological and parasitological high-sensitive tools are performed in endemic areas, almost one-third of the population have been reported to be affected by this liver fluke [5,6]. Even though most patients are asymptomatic, symptoms may be related to the acute infection (fever and abdominal pain) or to the chronic infection (biliary colic, cholecystitis and cholangitis) [7]. However, there is a paucity of studies that evaluate the natural history of subjects infected with fascioliasis (chronic inflammation, liver fibrosis stages, and carcinogenesis) and in those who were treated (post-infectious liver damage). Therefore, the long-term of fascioliasis are unknown.

Additionally, several studies reported an association between fascioliasis with other hepatic complications such as liver fibrosis, cirrhosis, and possibly also with cancer [8]. Due to these gaps in current knowledge regarding the natural history of fascioliasis, the aim of this study was to systematically review the literature to assess the role of *F*. *hepatica* in liver fibrosis, liver cirrhosis, and cancer.

## Materials and Methods

### Literature search

One of the authors (CM) designed and conducted the electronic search. We searched electronic databases to identify relevant studies (PubMed, LILACS, Scopus, Embase, Cochrane, and Scielo) from their inception through July 2015. The electronic search strategy was as follows: parasite (*Fasciola hepatica*) AND associated conditions (liver fibrosis, cirrhosis, tumor, cancer, neoplasia, malignancy, hepatocellular carcinoma, cholangiocarcinoma) [MeSH] AND associated terms (oncogene). The search term was adapted to the predominate language of the database. To identify additional candidate studies, we reviewed the reference lists of the eligible primary studies, narrative reviews, and systematic reviews. The search was conducted in accordance with the Preferred Reporting Items for Systematic Reviews and Meta-Analyses (PRISMA) guidelines [9].

### Screening and eligibility criteria

During the screening process, two reviewers working independently (CM, LAM) considered the potential eligibility of each of the abstracts and titles that resulted from executing the search strategy. We considered papers available in the following languages: English, Spanish, Italian, French, German, Turkish, Korean, Chinese, and Japanese. Those eligible publications were related to a possible direct relationship between fibrosis, cirrhosis, or cancer of the liver and presence of *F*. *hepatica*. During the elegibility process, all eligible studies based on their abstracts were then reviewed in full text versions. After reviewing the studies in full detail, we divided them into two groups: group 1, relevant and group 2, irrelevant. Relevant studies were those related to a possible direct relationship between the exposure and the disease. Irrelevant publications did not show a direct effect between the exposure and the disease. For example, a direct effect was when a parasite was found in liver fibrosis or cancer tissue, whereas an indirect effect (irrelevant) was if the parasite was mimicking a tumor without malignancy cells in the tissue. Any disagreement was resolved after consensus among all authors.

### Exclusion and inclusion criteria

**Fig 1:**
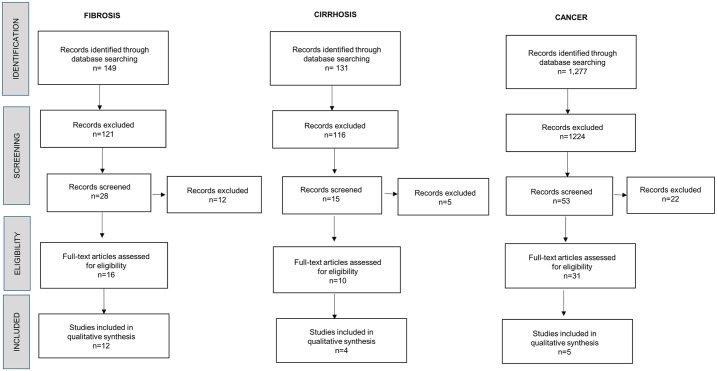
Flow diagram for selection of studies included in the review.

For clinical studies, articles were selected based on the evidence (pathological assessment and/or images) of *Fasciola* on fibrosis, cirrhosis or cancer of the liver. For basic research studies, articles were selected considering any evidence of fascioliasis and genetic alteration events, either *in vivo* or *in vitro*. Those that met the inclusion criteria were included in the final analysis and discussion (included studies, Fig 1).

### Analysis

Our analysis included those publications reporting fibrosis, cirrhosis as well as malignancy and identification of *Fasciola* either with direct or not relationship with fibrosis or tumor. Accordingly, any case report of liver fibrosis or malignancy was included in analysis only if a) shows a direct relationship with fascioliasis, b) occurs as a consequence of previous fascioliasis, or c) identification of the parasite in the tumor. Any reference meeting one of such criteria, whether national or international, was recorded, regardless of article type or quality.

### Data extraction

Data were then entered in a database covering: title, principal author, year of publication, type of study, method, results, and any additional comments. Details about the eligible studies are shown in S1 Table.

### Assessment of bias

Quality of studies was not used as a criterion to select or deselect the studies. Given that the primary outcome of interest was only to assess any relationship between *Fasciola* and the occurrence of liver fibrosis, cirrhosis or cancer; we did not explore the possibility of publication bias.

## Results

### Study selection

Our search strategy allowed us to identify 1,557 papers, of which 1,461 were excluded by title, abstract evaluation, and duplication. Duplicate entries were identified by considering the author, the year of publication, the title of the article, and the volume, issue and page numbers of the source. In questionable cases, the abstract texts were compared. As a result, 96 studies were initially screened by reviewing the corresponding full-text papers. Then, 39 records were excluded due to lack of consistent evidence related to cancer, fibrosis or cirrhosis. Thus, 57 records were then assessed for eligibility according to the criteria outlined previously. Finally, 21 were eligible for our analysis. Type of source included 100% journal manuscripts.

The included studies in the final analysis were divided into three categories based on the tissue effect gathered by *F*. *hepatica* exposure. Those three categories of publications were: (i) those publications related to the presence of *F*. *hepatica* and development of fibrosis; (ii) those publications showing data related to *F*. *hepatica* and consequent cirrhosis; (iii) those publications containing evidence of presence of *F*. *hepatica* and diagnosis of any cancer related to liver or biliary system. Based on such classification, we obtained a total of 1227, 149 and 131 studies in the first, second and third categories, respectively (Fig 1). As mentioned above, 21 full-text papers were eligible for final analysis. Five were selected as related to *F*. *hepatica* and cancer, 12 were related to *F*. *hepatica* and fibrosis, and 4 were related to *F*. *hepatica* and cirrhosis.

Information describing the studies analyzed are summarized in S1 Table, including author, publication year, study design, country, outcome, and evidence. We identified a total of 2 case reports and 2 case series (19.1%), 8 *in vitro* studies (.38.1%), and 9 animal assay studies (42.8%). The publication year ranged from 1974–2015. Geographically, 9 studies were conducted in Europe (42.9%), 9 in the Americas (42.9%), 2 in Asia (9.5%), and 1 in Oceania (4.8%).

### Fascioliasis and liver fibrosis

A total of 12 studies that reported liver fibrosis caused by *F*. *hepatica* infection (6 in animals, 4 *in vitro* studies, one human case series, and one human case report) were selected for this systematic review. A total of 3234 animals have been reported to have concomitant liver fibrosis and fascioliasis including bovine, sheep, calves, and pigs [10–21]. The first report of *Fasciola* and liver fibrosis was in 1977. Sheep infected with *F*. *hepatica* had fibrous tissue surrounding the hepatic lobules [12]. A higher number of parasites have a direct relationship with the degree of liver fibrosis in cattle (n = 10) [13]. In 3021 pigs, fascioliasis causes serious hepatic lesions mainly characterized by severe fibrosis [14]. In addition, periportal fibrosis and collagen deposition around the bile ducts has been also reported in cattle [15,16]. The more chronic injury around the bile ducts, the greater the periportal fibrosis. In an experimental animal model, the presence of collagen fibers around the bile ducts and cirrhosis with necrotic foci were found at 7 and 10 weeks post-infection with *F*. *hepatica*, respectively [17]; but the fibrosis may be reversible after effective anti-parasitic therapy. Whether liver fibrosis is entirely caused by Fascioliasis or by the host immune response is an open question not yet elucidated.

Among *in vitro* studies, some identified the gene expression patterns of animals infected with *F*. *hepatica* [18,19]. Up-regulation of fibrosis-related genes in *F*. *hepatica*-infected rats including collagen I, alpha-smooth muscle-actin, platelet-derived growth factor beta receptor, tissue inhibitor of metalloproteinase II, and activated human stellated cells (HSCs), have been reported [18]. This suggests that this parasitic infection may be associated with the development of liver fibrosis by activating the HSCs, similar to other infections (i.e. hepatitis C viral infection). In infected sheep, microscopic analysis of the livers showed massive infiltration of inflammatory cells and deposition of collagen at 8-week post-infection [19]. In addition, the authors reported up-regulation of genes associated with fibrosis (including genes in the JAK-STAT pathway), tissue repair, remodeling and regeneration (including *TNF-α*, *TGF-β*, *calponins*, *transgelins*, *osteopontin* and *adora2b*) [19]. Inoculation of native GST of *F*. *hepatica* in goats caused portal fibrosis, inflammatory infiltration with plasma cells, formation of lymphoid follicles, accumulation of haemosiderin-laden macrophages and granulomatous foci [20]. Natural killer cells have been also found in infected rats around the portal space, centrilobular veins, periportal fibrosis areas and around collagen [21].

In human studies, one human case with severe fascioliasis was reported to have fibrosis of portal tracks with fibrosis extending into the parenchyma after a liver biopsy [10]. The other study included 87 patients with fascioliasis and aimed to characterize by imaging the long-term liver damage after effective anti-parasitary treatment. This showed that 9 patients continued having fibrotic liver lesions after 1 year of treatment, but neither cirrhosis nor cancer was documented on any of these patients during a mean follow-up period of 62 months [11].

### Fascioliasis and cirrhosis

Four records were selected that reported cirrhosis as a concomitant condition of fascioliasis, including 3 *in vivo* studies and 1 case report. Two additional studies reported sequential progression from liver fibrosis to cirrhosis in animals infected with *F*. *hepatica* [13,17].

Liver cirrhosis has been reported in wild animal models including cows, goats and alpacas [13,22,24]. Cirrhosis in fascioliasis has been also reported in experimental animal models [17,23]. The liver damage reported in animals infected -for at least 6 months (chronic infection)- were described as fibrotic nodules (stage IV of liver fibrosis or cirrhosis) in most of the liver with several degrees of inflammation [22,24]. In 25 goats infected by *Fasciola* at least for 3 months, the authors reported loss of the lobular pattern, proliferation of bile ducts and fibrosis in portal areas consistent with cirrhosis after examining both lobes of the liver [22]. In another study of 40 infected rats, a pool of their bile ducts was collected and it was found that collagen I and III were significantly increased when compared to controls [23]. This was found similar to what occurs in cirrhosis.

In humans, one case has been reported with cirrhosis [25]. This was a 42-year-old American woman with fascioliasis who had an ERCP for biliary cirrhosis and cholangiogram suggesting sclerosing cholangitis [25]. The authors stated that the most direct cause for the primary biliary cirrhosis and other biliary complications on this patient was likely to be all caused by *F*. *hepatica*.

### Fascioliasis and cancer

A total of 5 studies were included in our systematic review. Four were *in vitro* animal studies, and one was an animal case series. We did not find reports of human cases of cancer explained by *Fasciola*.

Chung (2012) investigated the role of TGF-β and IL-4 in the immunosuppression as a hypothetical mechanism of parasite evasion of host immune system [26]. The study demonstrated that TGF-β and IL-4 are up-regulated as a consequence of *F*. *hepatica* infection, TGF-β reaches its maximum levels of serum at week 2 post infection in each mouse [26]. TGF-β is a potent known proliferation factor that can also directly inhibit activation of immune system [27]. Both increased proliferation by growth factors and immune evasion are cancer hallmarks [28]. Some studies conducted by Motorna (2011) and Gentile (1998) used the lambda/*lacI* Big Blue transgenic mouse model to investigate if genetic damage, as a measure of *lacI* mutations, could result in liver tissue from infection by *F*. *hepatica* [29,30]. There was an increase of *lacI* mutations in mice with fascioliasis suggesting that the infection increases the risk for complex hepatic cell mutations rather than mutations stemming from more definable oxygen radical-associated events [31,32]. An additional publication reported an indirect relationship between *F*. *hepatica* and cancer by induction of CYP2A5 enzyme (from the parasitic infection) which participates in the metabolism of carcinogens like B1 (AFB1) and several nitrosamines [32]. The results of this study suggested that *F*. *hepatica* can alter the activity of key hepatic enzymes, which may contribute to accumulation or decrease clearance of carcinogenic compounds found in food products or environmentally [32]. We found one publication that reported hepatocellular carcinoma (HCC) in cattle with fascioliasis [33].

Our results show a lack of findings/evidence of fascioliasis and cancer in population-based studies; quantitative data to measure association with liver disease (Odds Ratio, Relative Risk); publication bias; quantify significance with a funnel plot or Egger´s regression asymmetry test; long-term follow-up of infected cases to asses for further liver damage, and studies in human and animals.

## Discussion

As fascioliasis causes chronic infection in the liver, there is a need to elucidate the long-term clinical complications of this parasitic infection in humans. This systematic review summarizes the current evidence that may associate human fascioliasis with liver fibrosis, cirrhosis, and perhaps cancer.

**Fig 2:**
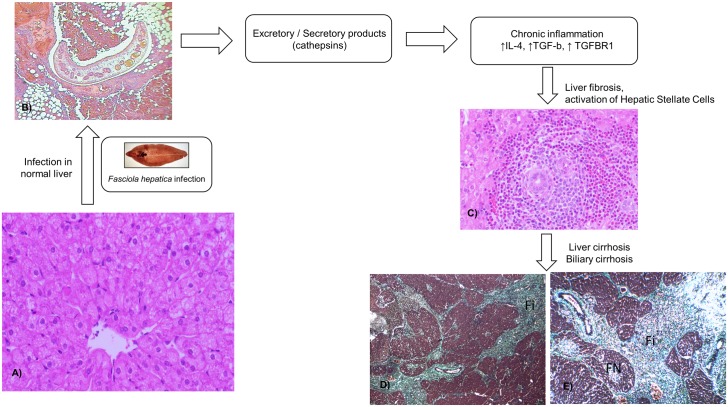
Schematic representation of liver fibrosis and cirrhosis associated with fascioliasis. (A) Normal architecture of a sheep's liver. Haematoxylin and eosin (HE) staining. Taken from Ref. [19]. (B) Juvenile parasite migrating in the peritoneal cavity causing destruction and haemorrhages in case (HE stain, magnification x100). The biopsy in the peritoneum was performed due to clinical suspicion of metastases Taken from Ref. [48]. (C) Typical microscopic appearance (400X) of liver from the infected sheep at 8 weeks post infection, massive infiltration of inflammatory cells and deposition of collagen can be observed. Taken from Ref. [19]. (D) Liver cirrhosis caused by *F*. *hepatica* infection. Trichrome stain (100x). Rat. Authors’ photo gallery. (E) Extensive fibrosis (Fi) with formation of fibrotic nodules (FN), architectural disruption of the liver and regeneration; stage IV or F4 (cirrhosis). Trichrome stain. Rats. Authors’ photo gallery.

We showed that *Fasciola* plays an important role in the development of liver fibrosis, and cirrhosis in animal models as shown in Fig 2 [10–21,34]. The mechanism of this association may be due to the activation of HSCs by the cathepsins of the parasite [18]. The intensity of infection may play a role on the development of liver fibrosis during the infection. In addition, there is weak evidence in the role of *Fasciola* in liver fibrosis in humans. This is limited to case reports. The impact of this association has not been well established in populations highly prevalent with *Fasciola*. It will be important to identify at a population level whether patients with *Fasciola* are more prompted to develop liver fibrosis and cirrhosis compared to non-infected patients, after controlling for alcohol consumption and viral etiologies. Furthermore, the role of early detection, and early treatment of acute or chronic human fascioliasis has not been studied yet as a strategy to prevent development of fibrosis overtime.

To further justify the plausibility that *F*. *hepatica* is associated with fibrosis, there are reports of fibrosis triggered by related organisms [34,35]. Livers of 35 fallow deer with fascioliasis, caused by the related parasite *Fascioloides magna*, were found to contain proliferation of the epithelium of bile ducts (biliary proliferation, an early stage for biliary cirrhosis) which were framed with a large quantity of connective tissue [35]. In that study, myofibroblasts especially HSCs were determined to play an important role in the synthesis of extracellular matrix components in the development of parasitic fibrosis and cirrhosis in the liver of these animals [35]. Similarly, fibrosis has been reported in 15 cows as a consequence of fascioliasis caused by *F*. *gigantica* with presence of proliferative and hyperplasic bile ducts [34].

In carcinogenesis, the evidence of the association of *Fasciola* infection and cancer is very limited and no conclusions can be unequivocally reached based on our findings. *Fasciola* infection in animal models has been demonstrated to overexpress a proliferative factor such as TGF-β, increase mutations (*lacI*) in mice, and induce CYP2A5 isoenzyme. The later may result in a reduction on the metabolism of carcinogenic agents. Our results remain informative since no animal (except for ref.33) or human studies have shown a case of cancer and *Fasciola*. Furthermore, the evidence of *Fasciola*-related cancer in animals or humans has not been documented but the reasons for this are beyond of the aim from this study. We can speculate that as most endemic areas of fascioliasis are located in resource-poor settings, where the access to healthcare centers is limited, the chronic consequences from the infection by *Fasciola* are likely underreported and therefore, unknown. For instance, there has been a study to suggest an early presentation of liver cancer in young adults in Peru, but no etiology has been identified [36]. A recent study on HCC patients in Peru showed that all four K-RAS-mutated HCCs were unusual I21M mutants, uncommon K-RAS mutations different from codon 12 mutations have been associated with cholangiocarcinoma produced by viral infections or fluke infestations [37,38]. To the best of our knowledge, there has not been any association between liver cancer in Peru and fascioliasis to date. *F*. *hepatica* is able to induce DNA damage through action of mutational mediators such as reactive nitric species and reactive oxygen species [39]. Both cirrhosis and genomic instability combined to a protumorigenic environment caused by CYP2A5 mutated contribute to cell transformation.

The results from this systematic review are interesting for several reasons. Most of the people chronically infected by *Fasciola* are asymptomatic but they are not necessarily free of inflammation [7]. There is a degree of inflammation into the liver in those chronically infected individuals by means of an increase in serum lipid peroxidation and a decrease in antioxidant enzymes, but the period between initial infection and developing of liver fibrosis in humans is still unknown [40]. Intensity of infection, length of infection, re-infections, other liver diseases, alcohol consumption, co-infection with chronic viral hepatitis, among others; are factors to be considered when assessing liver disease in infected individuals by *Fasciola* in endemic areas. For example, alcohol consumption can exacerbate cholangiofibrosis in hamsters infected by Opisthorchis, another liver fluke infecting bile ducts, but no studies in *Fasciola* have been performed [41]. One also might think that the inflammation from the infection would resolve after effective antiparasitic therapy but this has not yet been assessed. Future longitudinal studies in human from endemic areas may investigate further our findings. In addition, future studies may face the challenges of *Fasciola* resistant to triclabendazole, the only drug available for *Fasciola* nowadays [42].

The major limitation of our study is the absence of previously published population studies that assessed the role of *F*. *hepatica* in liver fibrosis, cirrhosis, and cancer. Therefore, our results come from basic science studies, animal models, case reports, and case series. However, no studies have previously systematically reviewed the literature in this important topic, and our study serves to suggest an association between *F*. *hepatica* with liver fibrosis and cirrhosis. Other limitations include publication bias, and lack of longitudinal follow-up of infected patients. Despite these limitations, we believe that our study makes an important contribution to recognize several potential severe chronic complications associated with human fascioliasis, and will be the base of future population studies that assess these associations. The results of our study and future studies will be of use for vulnerable populations affected by this fluke, in areas like the Peruvian Highlands, to prevent the complications caused by fascioliasis. Furthermore, it is relevant to investigate the existence of an inter-relationships between *F*. *hepatica* and gastrointestinal tract microbes that may affect the progression of fascioliasis. For instance, *Helicobacter pylori* infection has been closely associated with *O*. *viverrini-*associated cholangiocarcinoma suggesting that the liver fluke is a reservoir of the carcinogenic bacterium and thus making plausible that the co-infection may promote the pathogenesis of cholangiocarcinoma [43,44]. Additionally, studies of the biliary microbiota should reveal the role of host microbial content in the development of fascioliasis and whether alterations or changes in microbiota occur as a consequence of the liver damage. Most recently, compositional shifts in the tissue microbiome of *O*. *viverrini*-associated cholangiocarcinoma were identified suggesting that changes in the microenvironment occurred after parasite infection can trigger tumorigenesis [45].

Also, comparison of similarities and dissimilarities with the pathophysiological processes leading to liver cancer and cholangiocarcinoma, induced by infection with *O*. *viverrini* and *C*. *sinensis*, and including well-characterized liver fluke derived metabolites likely will advance this task [46,47]. We conclude that there is some evidence of an association between *Fasciola* infection with liver fibrosis and cirrhosis but no strong evidence between *Fasciola* and cancer. There is a need of long-term population studies to assess the association of *F*. *hepatica* with liver fibrosis, cirrhosis and cancer in endemic populations.

## Supporting Information

S1 Table Studies included in final analysis. (DOCX)
