# Supplementary material for: Association of Fasciola hepatica Infection with Liver Fibrosis, Cirrhosis, and Cancer: A Systematic Review
Source: PLoS Negl Trop Dis. 2016 Sep 28;10(9):e0004962. doi: 10.1371/journal.pntd.0004962 (PMC5040415; doi:10.1371/journal.pntd.0004962)
Supplement: S1 Table — (DOCX) [file pntd.0004962.s001.docx]

**S1 Table. Studies included in final analysis.** List of 21 publications considered in final analysis grouped by the type of liver damage associated with Fascioliasis including fibrosis, cirrhosis and cancer.

| **Author** | **Year** | **Study Design** | **Sample** | **Country** | **Outcome** | **Evidence/Method** | **Ref** |
| --- | --- | --- | --- | --- | --- | --- | --- |
| **FIBROSIS** | | | | | | | |
| Alvarez | 2015 | *In vitro* | 8 | Australia | Hepatotoxicity and fibrosis | Fibrosis-related gene expression | 19 |
| Marcos | 2011 | *In vitro* | 18 | Peru | Liver fibrosis | Fibrosis-related gene expression | 18 |
| Zafra | 2010 | *In vitro* | 18 | Spain | Granulomas and fibrosis | Microscopy | 20 |
| Tliba | 2002 | *In vitro* | 12 | France | Periportal fibrosis | Immunohistological staining | 21 |
| Kabaalioglu | 2007 | Case series | 87 | Turkey | Liver fibrosis | Imaging (sonography and CT) | 11 |
| Jones | 1977 | Case report | 1 | England | Biliary obstruction, granulomas, patchy periductular fibrosisury | Microscopy | 10 |
| Kolodziejczyk | 2015 | *In vivo* | 12 | Poland | Fibrosis and cirrhosis in the liver | Increased expression of cytokeratins | 17 |
| Trivilin | 2014 | *In vivo* | 100 | Brasil | Liver fibrosis | Histopathology | 16 |
| Capucchio | 2009 | *In vivo* | 3021 | Italy | Liver fibrosis | Electron microscopy | 14 |
| Marcos | 2007 | *In vitro and in vivo* | 24 | Peru | Fibrosis and cirrhosis in the liver | Histopathology | 13 |
| Wensvoort | 1982 | *In vivo* | 10 | Netherlands | Liver fibrosis | Histopathology | 15 |
| Rushton | 1977 | *In vivo* | 11 | England | Parenchymal and biliary fibrosis | Microscopy | 12 |
| **CIRRHOSIS** | | | | | | | |
| Timoteo | 2005 | *In vivo* | 8 | Peru | Liver necrosis and cirrhosis | Immunohistochemistry, increase of liver SGOT and SGPT | 24 |
| Perez | 1999 | *In vivo* | 25 | Spain | Severe hepatic damage, cirrhosis | Histopathology and immunohistochemistry | 22 |
| Mark | 1983 | *In vivo* | 100 | USA | Liver cirrhosis | Collagen I and III | 23 |
| Hauser | 1984 | Case report | 1 | USA | Secondary biliary cirrhosis, and pancreatitis | ERCP | 25 |
| **CANCER** | | | | | | | |
| Chung | 2012 | *In vitro* | 30 | Korea | Splenomegaly | Increased levels of TGF-β and IL-4 | 26 |
| Motorna | 2001 | *In vitro* | 64 | USA | Liver damage | Parasite-induced mutations | 29 |
| Montero | 1999 | *In vitro* | 22 | USA | Liver injury | Increased activity of the CYP2A5 isozyme | 33 |
| Gentile | 1998 | *In vitro* | 13 | USA | Liver damage | Increased genetic damage | 30 |
| Vitovec | 1974 | Case series | 279 | Germany | Malignant transformation | Histopathology | 33 |
